# A variable-stiffness tendril-like soft robot based on reversible osmotic actuation

**DOI:** 10.1038/s41467-018-08173-y

**Published:** 2019-01-21

**Authors:** Indrek Must, Edoardo Sinibaldi, Barbara Mazzolai

**Affiliations:** 1 Center for Micro-BioRobotics, Istituto Italiano di Tecnologia (IIT), Viale R. Piaggio 34, 56025 Pontedera, Italy; 2 Institute of Technology, University of Tartu, Nooruse 1, 50411 Tartu, Estonia

## Abstract

Soft robots hold promise for well-matched interactions with delicate objects, humans and unstructured environments owing to their intrinsic material compliance. Movement and stiffness modulation, which is challenging yet needed for an effective demonstration, can be devised by drawing inspiration from plants. Plants use a coordinated and reversible modulation of intracellular turgor (pressure) to tune their stiffness and achieve macroscopic movements. Plant-inspired osmotic actuation was recently proposed, yet reversibility is still an open issue hampering its implementation, also in soft robotics. Here we show a reversible osmotic actuation strategy based on the electrosorption of ions on flexible porous carbon electrodes driven at low input voltages (1.3 V). We demonstrate reversible stiffening (~5-fold increase) and actuation (~500 deg rotation) of a tendril-like soft robot (diameter ~1 mm). Our approach highlights the potential of plant-inspired technologies for developing soft robots based on biocompatible materials and safe voltages making them appealing for prospective applications.

## Abstract (web summary)

Plant-inspired osmotic actuation has been proposed as a competitive actuation strategy yet reversibility is still an open issue hampering its implementation in soft robotics. Here the authors show a low input voltage reversible osmotic actuation strategy based on the electrosorption of ions on flexible electrodes.

## Introduction

Soft robots are rapidly gaining momentum also owing to their intrinsic material compliance to enhance interaction with delicate objects^1^, humans and unstructured environments^2^. However, irrespective of longer-term applications, the complex interplay between material and geometrical properties underlying their design still poses fundamental scientific problems. In fact, a major issue is the quest for actuation strategies seamlessly integrated in a soft body, intrinsically associated with stiffness modulation^3^, also functional to an effective interaction with the environment^4^. Fluid-based strategies provide a natural option: combustion, e.g., was demonstrated in a soft robot made through a multimaterial three-dimensional printing^5^, whereas the catalytic decomposition of an on-board fuel supply was integrated in an autonomous soft robot constructed through molding and soft lithography^6^. Living actuation strategies represent an extraordinary source of inspiration to generate solutions intrinsically soft and safe. In this regard, complementary takes focused on multifunctional materials, including plant-inspired hygromorphic bilayers^7,8^ and anisotropically patterned hydrogels^9^ capable of morphing their shape exposed to humidity.

Indeed, water transport in plants provides a remarkable source of inspiration for the development of soft actuation strategies, also because non-hazardous fluids can enable applications effectively interfaced with humans. Plant cell turgor (pressure) is generated by water influx due to osmolyte concentration gradients through the cell wall and plasma membrane, which behave as an osmotic barrier. Moreover, plants use the coordinated modulation of intracellular turgor to tune their stiffness and achieve macroscopic movements^10^. The cytosolic osmolyte system is an aqueous gel solution containing proteins and small molecules (including potassium chloride, glucose, and glutamine)^11^, and plant cells actively control osmolyte gradients (also using transmembrane ion-pumping proteins^12^) to generate reversible movements. Indeed, the osmolyte transport across the cell boundary locally creates differences in concentration between the extracellular fluid (ECF) and the intracellular fluid (ICF), which translates into an osmotic pressure difference *Π* = *Π*_ICF_−*Π*_ECF_. Given the (hydrostatic) pressure difference *P* = *p*_ICF−_*p*_ECF_, the water flow rate (taken as positive towards the ICF) is then driven by *Π*−*P* (up to a scaling factor related to the osmotic barrier surface and permeability, and by neglecting correction factors accounting for osmolyte rejection)^10,11^, and the resulting turgor trend also depends on the elastic properties of the cell wall confining the volume variation^10^. Reversibility is then achieved by controlling osmolyte concentration in a cyclic fashion.

Plant-inspired osmotic actuation was recently proposed as a competitive strategy^13^. Starting from osmotic actuation modeling^14^, a plant-inspired actuator^13^ was demonstrated (characteristic size ~1 cm): using sodium chloride as osmolyte, our device produced ~20 N force in ~2 min when using a 2 M initial osmolyte concentration. However, the actuation trend was dictated by the initial osmolyte concentration. Hence, reversibility was still an open issue hampering its implementation, also in soft robotics.

In this study we show a reversible osmotic actuation strategy based on the electrosorption of ions on flexible electrodes (made of non-hazardous material, such as porous carbon), driven at low input voltages (1.3 V). By intertwining principles from osmosis-based plant actuation and capacitive water desalination^15^, we design an electroactive unit (characteristic size ~1 cm) that reversibly controls the concentration of ions (acting as osmolyte) obtained through the dissolution of an electrolyte (sodium sulfate) in water. We then demonstrate reversible stiffening (~5-fold increase) and actuation (~500 deg rotation) of a tendril-like soft robot (diameter ~1 mm) coupled to the control unit. The modular design proposed can be extended to a variety of soft robot effectors. Considering the integrated design of our miniature actuator, as well as the use of biocompatible materials and safe voltages, osmotic actuation could meet the size/safety requirements of further prospective applications^2^.

## Results

### Osmotic actuation working principle and soft robot concept

**Fig. 1:**
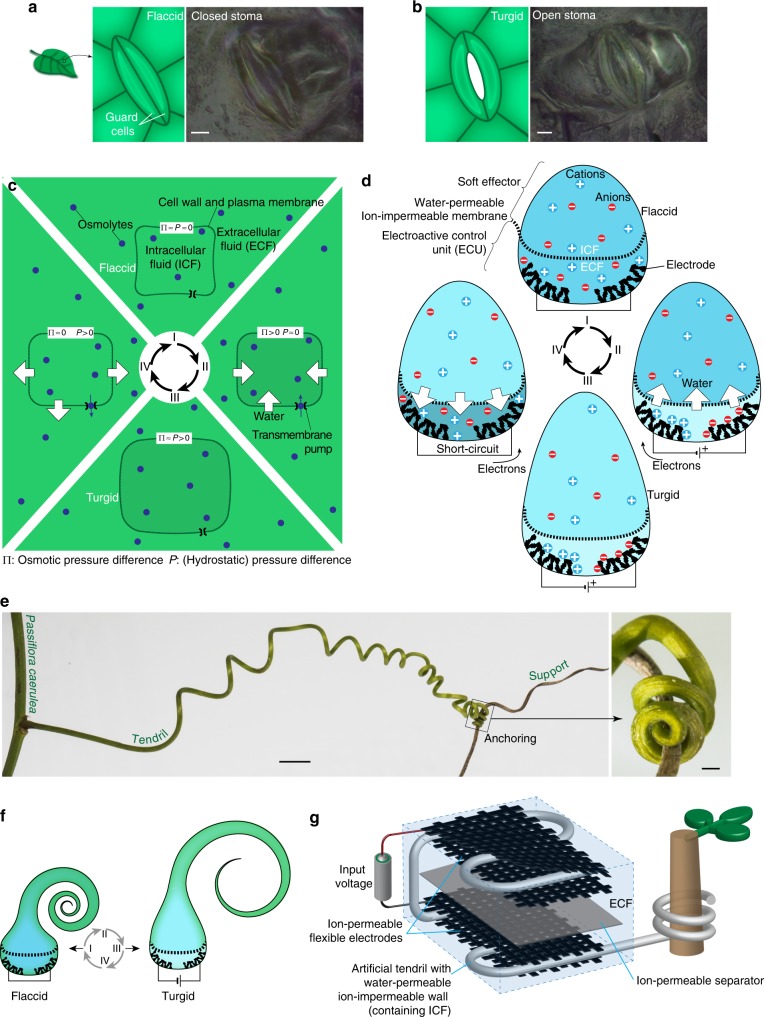
Osmosis-based reversible actuation: from plant cell to a tendril-like soft robot. **a**, **b** Stomata guard cells in **a** flaccid and **b** turgid state: schematic and optical images of *Tradescantia zebrina* leaves (courtesy of Andrea Degl’Innocenti). Scale bars: 10 µm. **c**, **d** Cyclic osmoregulation (schematic) **c** for plant cell and **d** for our osmotic actuator. The concentration of ions (osmolyte) was controlled in the electroactive control unit through the reversible immobilization on the surface of electrodes, as driven by an input voltage (ions are released to the fluid bulk upon short-circuiting). Ion concentration modulation drives the water flux as in plant cells, and modulates the soft effector stiffness. **e** *Passiflora caerulea* tendril reaching and anchoring to an external support. Scale bars: 5 mm main image, 0.5 mm detail. **f** Artificial tendril actuation (schematic). **g** Tendril-like soft robot based on the osmotic actuator (schematic)

Starting from reversible plant movements such as the opening/closure of the stomata involved in leaf transpiration and gas exchange with the atmosphere (Fig. 1a, b), we schematized cyclic osmoregulation as in Fig. 1c, whence we derived the working principle shown in Fig. 1d. Ions were adopted as osmolyte, obtained by dissolving an electrolyte in water. Ion concentration was controlled within an electroactive control unit (ECU), separated from a soft effector by a water-permeable ion-impermeable membrane. Electrosorption was used to control ion concentration: by applying a voltage, ions were immobilized on the surface of flexible electrodes introduced in the ECU; by short-circuiting, ions were released back to the fluid bulk. The osmotic pressure difference, created through electrosorption, induced a water flux between the ECU and the soft effector (the electrolyte filling both the soft effector and the ECU plays the role of ICF and ECF), and reversible actuation was achieved by reversibly operating the input voltage. The functional analogy between the osmoregulation in plant cells and in our osmotic actuator is illustrated through the corresponding states (I–IV) in Fig. 1c, d.

An illustrative embodiment was introduced for the effector drawing inspiration from climbing plants^16,17^, specifically from the organs that some have developed to come into contact with and climb neighboring objects, i.e., tendrils^17,18^. While growing, they are able to reach and anchor to external supports, and to coil along their length so as to shorten and hoist the plant toward the anchoring point (Fig. 1e). This complex movement is not purely osmotic (coiling, e.g., is due to the asymmetric contraction of an internal fiber ribbon of specialized cells^18^). Some tendrils feature a hook-like tip structure that clips-on-clips-off onto supports like a carabiner: also based on contact cues, they anchor by coiling around the support (and thicken and lignify, so that also this movement is not purely osmotic)^16,17^. Nonetheless, a simplified osmosis-driven uncoiling/coiling of an artificial tendril was considered (Fig. 1f). In our embodiment (Fig. 1g), the electrodes (surrounded by, and accommodating the electrolyte solution to functionally obtain the ECU) were put in contact with a water-permeable ion-impermeable section of the tendril wall acting as an osmotic membrane. Supplementary Movie 1 illustrates the proposed concept.

### Osmolyte concentration modulation by electrosorption

**Fig. 2:**
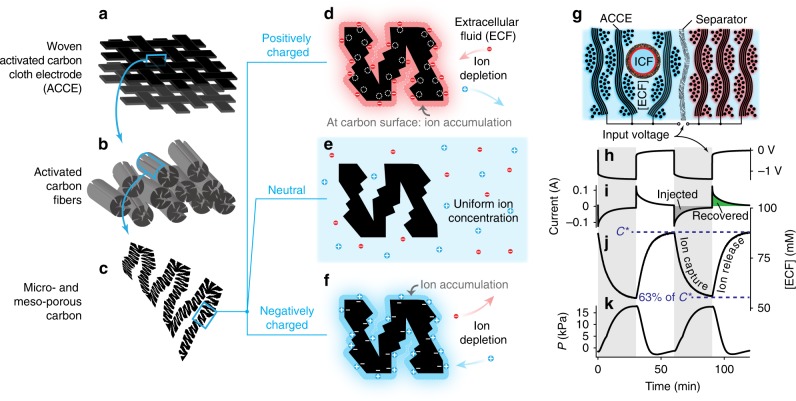
Electrosorption as a key mechanism to reversibly modulate osmolyte concentration. **a**–**c** Hierarchical structure of the activated carbon cloth electrodes (ACCEs): **a** textile woven from **b** bundles of binderless fibers provide ion passage pathways, whereas **c** porous carbon provides a large specific surface area enhancing ion electrosorption capacity. **d**–**f** Close-up of **d** positively charged, **e** neutral, and **f** negatively charged ACCE. **g** In situ measurement (schematic) of ion concentration ([ECF]) and pressure (*P*). **h** Input voltage (1.3 V). **i**–**k** Measured **i** current, **j** concentration and **k** pressure difference, using sodium sulfate (Na_2_SO_4_) as electrolyte. The measured trends highlight a reversible behavior; the colored areas in (**i**) illustrate that 90% of the initially injected charge was recovered

High-specific-surface-area activated carbon cloth electrodes (ACCEs) were selected which, being also marketed as wound dressing, feature flexibility, and biocompatibility. Their hierarchical structure of binderless carbon material (Fig. 2a–c) is favorable for hosting the ECF, thus for creating ion pathways in-between the carbon fibers. Their meso-porous and micro-porous structure yields a high areal capacitance, suitable to store a considerable amount of charges: the injected electronic charge is balanced by counter-ions that accumulate from the ECF bulk onto the ACCE surface (electric double layer formation) (Fig. 2d–f). This, in turn, induces ion depletion in the ECF bulk (as also proposed for water desalination^15^) and builds the osmotic pressure gradient driving the water flux and thus actuation. The ion concentration and pressure generation control were assessed by driving the ECU with 1.3 V voltage (Fig. 2g–k). In particular, the osmotic membrane (polyamide film deposited on the inner wall of a porous polysulfone tube) was placed in-between the layers of an ACCE (Fig. 2g), at the location where the largest concentration modulation^19^ is expected (leading to the formation of the largest osmotic pressure gradients). Sodium sulfate (Na_2_SO_4_) was chosen as electrolyte, at a low (0.1 M) concentration, because of its very low toxicity yet high conductivity and electrochemical stability. Electrosorption turned out to be an energy-effective mechanism to achieve a reversible control: e.g., upon short-circuiting, 90% of the initially injected charge was recovered.

### ECU pressure generation performance

**Fig. 3:**
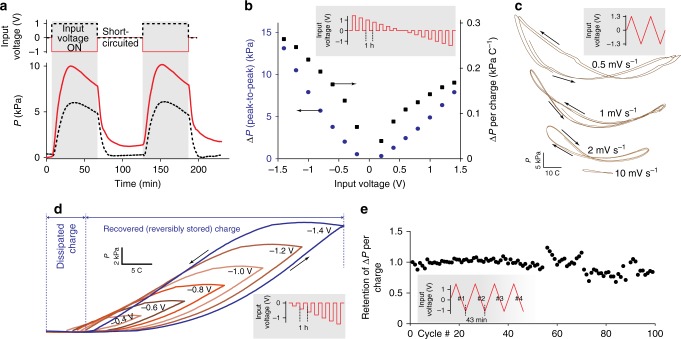
Pressure generation performance of the electroactive control unit. **a**–**c** Pressure trends showing the effect of polarity: **a** pressure (*P*) vs. time; **b** pressure variation (Δ*P*, peak-to-peak) vs. input voltage; **c** pressure generation vs. charge (obtained by integrating the current to/from the electrodes) for selected values of the voltage scan rate. **d** Charge recovery when returning to the initial, uncharged state, demonstrating good energy-efficiency. **e** Performance retention over cyclic operation: the generated pressure per consumed charge retained ~85% of its initial value after 100 cycles

Based on the configuration sketched out in Fig. 2g, the ECU performance was extensively characterized. A polarity-dependent behavior was observed (Fig. 3a–c). In particular, an approximately two-times-higher turgor (*P*) was obtained when inserting the osmotic membrane in the negatively charged ACCE (Fig. 3a), likely due to presence of charged surface groups such as carboxyl groups on the ACCE surface. The peak-to-peak pressure variation (Δ*P*) linearly increased with the input voltage (Fig. 3b), remarking the dominantly capacitive nature of the ECU. Also the fact that higher voltages yielded higher charge efficiency, expressed as a relatively larger Δ*P* per charge, provided similarities with capacitive deionization^15,20^. The polarity-dependent character of the turgor formation—further observed through a dynamic characterization (Fig. 3c)—is reported in this study (polarity-dependent trends of porous carbon electrodes were also reported by previous studies addressing actuation through a different approach, namely through a volumetric expansion^21,22^). Based on the measured charge-recovery trends (Fig. 3d), our electroactive control potentially allows to reuse most of the charge and energy through subsequent actuation cycles. Besides promoting energy-efficiency, this aspect prospectively supports the development of applications also based on a concurrent energy storage. Indeed, a good performance retention was measured: e.g., the generated pressure per consumed charge retained ~85% of its initial value after 100 cycles (Fig. 3e).

### Tendril-like soft robot

**Fig. 4:**
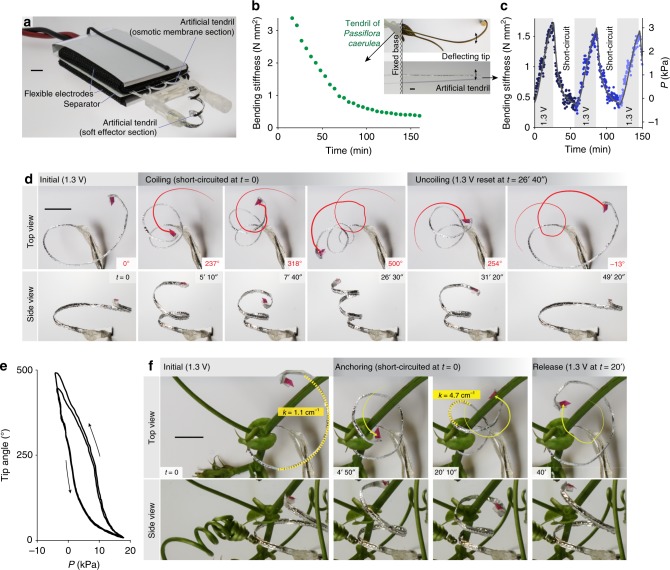
Reversible stiffness modulation and actuation of a tendril-like soft robot. **a** Tendril-like soft robot based on the osmotic actuator. A section of the tendril (the one snaking in-between the ACCEs layers) acts as osmotic membrane, another provides the soft effector. **b**, **c** Bending stiffness vs. time. **b** for a cut tendril of *Passiflora caerulea* (turgor decrease caused by dehydration), and **c** for the soft robot (reversible stiffness modulation, also correlated to turgor *P*, driven by the input voltage). **d** Tip rotation angle at selected times: the soft robot reversibly spanned ~500 deg over ~25 min. **e** Tip rotation angle vs. turgor, showing good repeatability. **f** Strong (~5-times) change in curvature (*k*) featured by the soft robot during reversible coiling (around a *Passiflora caerulea* stem). Scale bars: 5 mm

A tendril-like soft robot (~1 mm diameter) was built by connecting two tubular sections: one (based on the aforementioned polysulfone tube) snaking in-between the ACCEs layers and acting as osmotic membrane, the other (made of polyethylene terephthalate) acting as effector (Fig. 4a). The bending stiffness of the artificial tendril was of the same order as that of a *Passiflora caerulea* tendril (Fig. 4b, c). Similarly to the natural tendril, for which a decrease in turgor induces a corresponding reduction in stiffness (Fig. 4b), the soft robot stiffness was modulated: by controlling the input voltage, an approximately five-fold reversible increase in bending stiffness was demonstrated (Fig. 4c). Inspired by the osmosis-driven movement of hook-like tendril structures around their support^16,17^, a reversible actuation through uncoiling/coiling cycles was assessed. The tip angle of the artificial tendril reversibly spanned ~500 deg (Fig. 4d) over a timescale comparable to that of natural tendrils (1 h to complete coiling^23^), also showing good repeatability (Fig. 4e). Natural tendrils often require the presence of a support in order to increase their stiffness and to maintain prolonged contact. The strong (~5-times) change in curvature associated with the considered soft robot actuation (Fig. 4f) can be used for hooking/anchoring tasks, similarly to natural tendrils. Supplementary Movie 2 illustrates the reversible actuation of the tendril-like soft robot.

## Discussion

In contrast to phenomenological imitation (as obtained, e.g., when mimicking skeletal muscles through the direct volumetric expansion of the electrodes^24,25^), our osmotic actuator mimics plants also structurally and functionally. A highly-integrated actuator was obtained owing to structural cues (osmotic membrane at the ECF|ICF interface, geometry/material interplay) and functional cues (active osmoregulation) drawing inspiration from plants. Indeed, on the basis of their hierarchical composite structure made of multifunctional materials, plants can provide a model for soft robots.

Building on the potential of osmotic processes for actuation^13,14,26,27^, our integrated design leverages an electrosorption-based in situ osmolyte concentration control to achieve reversibility, and can be amenable for miniaturization since it does not require off-board pumps. Our actuator, having a characteristic size ~1 cm, featured a characteristic time on the order of 10 min. Previous models^13,14^ showed how the actuation performance scales with the geometrical and material properties of the actuator: those models should be extended so as to also include electrosorption-driven ion dynamics (both in the bulk liquid and in porous media) in order to predict the performance of our actuator. Furthermore, we reported stiffness variation in response to a single step voltage input (Fig. 4c). Prospectively, additional studies could demonstrate continuous stiffness control in a range by a closed-loop strategy. Indeed, based on the findings of this study, the control feedback could be based on ion concentration (Fig. 2j) or input charge (Fig. 3b). Moreover, the proposed osmotic actuation also uses flexible biocompatible materials and safe physico-chemical processes. Hence, it could provide a valuable option for prospective applications where size/safety constraints play a role^2^. Subsequent embodiments could further integrate structural and functional cues, e.g. by sewing the osmotic membrane section of the artificial tendril onto the ACCEs, thus paving the way for systems with enhanced flexibility.

To conclude, our approach highlights the great potential of plant-inspired technologies^4,13,14,28–30^, to improve morphological, chemical and functional adaptation of soft robots to the working environment. Indeed, concurrently addressing powering, actuation and control by following a bioinspired approach, leads to an integrated design that can effectively enable the development of soft robots^6,31^ and push the frontier of robotics science per se^2,4^.

## Methods

Two embodiments were considered, with the osmotic membrane snaking in-between either one (Supplementary Fig. 1a-b) or two (Supplementary Fig. 1c-d) ACCEs. The configuration in Supplementary Fig. 1a-b was used to extensively characterize the ECU performance (see Figs. 2 and  3) by considering a single ACCE (as typical in electrochemistry). Based on the said characterization, we then adopted the configuration in Supplementary Fig. 1c-d to build and characterize the soft robot (see Figs. 1 and 4); indeed, the latter configuration allows for a greater water exchange between the ECU and the effector, thus enhancing the actuation performance. The fabrication process is identical for both configurations.

All chemicals were used without further purification or activation.

All the experiments were performed at 25 ± 1 °C unless otherwise specified.

### ECU fabrication

As regards the ACCEs, plain weave activated carbon cloth (ACC) (Zorflex FM10, Calgon Carbon) with ~0.5 mm thickness and with ~70 Ω square^−1^ sheet resistance in dry was used as electrode material. A cellulose-based filter paper (75 g m^−2^, Carlo Erba) separator was inserted between two identical electrodes, either having 47 cm^2^ active area in three layers (Supplementary Fig. 1b,d). Electronic connection between the monolithic ACC fibers and the copper connecting wires was achieved using silver-loaded conductive paint (RS Pro, RS components); the electrical connection was hermetically sealed from electrolyte access using silicone (Henkel AG) (Supplementary Fig. 2a). The electrodes were then loaded with 0.1 M sodium sulfate (>99.5%, Fischer) aqueous solution (acting as ECF). The electrodes accommodated ~3.3 g g^−1^ of ECF by capillary force. Possible irreversible ion absorption by ACC upon first immersion with the electrolyte solution was compensated by exchanging the ECF solution at least five times before measurements.

As regards the osmotic membrane section of the artificial tendril, porous polysulfone hollow fiber membranes with 0.5 mm diameter, rated at 50 kD or 0.05 μm pore size (MicroKros, Spectrum Labs), were used as substrates for the osmotic membrane (Supplementary Fig. 1b,d). The hollow fibers were received as dry and they were first fixed to a serpentine shape using epoxy adhesive. The shaped hollow fibers were first conditioned by soaking in and flushing with 20% ethanol or isopropanol solution in water and then flushed with deionized (DI) water. Then, polyamide osmotic membrane was synthesized^32^ on the inner surface of the hollow fiber by first soaking the tube in an aqueous solution of 2 wt% m-phenylenediamine (99%, Sigma), 2 wt% (1R)-(−)-10-camphorsulfonic acid (98%, Sigma), 1 wt% triethylamine (≥99%, Sigma), and 0.2 wt% sodium dodecylbenzenesulphonate (99%, Acros Organics) for approximately 20 min and then flowing a solution of 0.2 wt% trimesoylchloride (98%, Sigma) in hexane (95%, Sigma) through the hollow fiber during 1 min at a rate of ~10 ml min^−1^. The finished composite fibers were stored while immersed in DI water.

The placement of the osmotic membrane on the inner surface of the hollow fiber mitigates the internal concentration polarization limitation known in pressure-retarded osmosis units^33–35^, as the electric-field-driven ionic current during the ECF concentration modulation is allowed to pass through the porous wall of the hollow fiber (but not to flow through the osmotic membrane itself), effectively avoiding formation of an unstirrable electrolyte volume within the porous wall.

The osmotic membrane section of the artificial tendril was filled with the aforementioned sodium sulfate aqueous solution (also acting as ICF), then it was placed in-between the first and the second ACCE layer, counting from the separator (Supplementary Fig. 1b,d), where faster ion concentration control is expected based on previous studies^19^. Positive polarity of the applied voltage refers to the ACCE surrounding the artificial tendril being positively polarized. The ECU assembly is shown in Supplementary Fig. 2b; the main assembly steps are recalled in Supplementary Movie 3.

### Conductivity probe for in situ concentration measurement

In situ concentration measurement (close to the osmotic membrane section of the artificial tendril) was performed using a custom low-profile conductivity probe (Supplementary Fig. 3a-c). In particular, two gold filaments (20 μm, Heraeus) were laminated in-between two 60 μm fused monofilament polyester meshes (12 threads mm^−1^, MooStar Printing), and connected through silver-loaded conductive paint to a frequency analyzer (REF600, Gamry). The active sensor area (1.5 mm characteristic size) was defined by casting the other areas into epoxy resin. The real part of impedance (*Z*_re_) between the gold filaments was first calibrated for sodium sulfate concentration (Supplementary Fig. 3d); the calibration solutions were soaked into pieces of ACC surrounding the conductivity probe. Then, the conductivity probe was inserted close to the artificial tendril. The input voltage to the ECU was supplied from a battery (size AA, Duracell) in series with a voltage-limiting diode (B340LA, Diodes Inc.) and a 2-Ω shunt resistor for current measurement (Supplementary Fig. 3e). The experiment was performed in a Faraday cage.

### ECU electrochemical characterization

Driving signals were generated and impedance was measured using an electrochemical workstation (REF600, Gamry) (Supplementary Fig. 4a). The current-collector-free ACCE demonstrated a predominantly capacitive behavior (double layer capacitance *C*_a_ = 420 mF cm^−2^) and showed remarkably low values for real impedance (*Z*_re_), below 11 Ω at all frequencies (Supplementary Fig. 4b). The electrical impedance of the uncharged ECU assembly showed a pronounced decrease when increasing the electrolyte concentration, suggesting a good match between the ACCE impedance (*R*_ACCE_) and the electrolyte impedance (*R*_E_). Electrolyte solution dilution by charging the ECU assembly to open-circuit voltage of 0.87 V decreased the real impedance by 7.7% (Supplementary Fig. 4c). This implied a decrease in *R*_ACCE_ due to the increased ionic and electronic charge density at ACCE surface, even dominating over the ion depletion in the ECF bulk.

Bipolar cyclic voltammetry was performed (Supplementary Fig. 4d). The ACCE differential specific (i.e. gravimetric) capacitance at 0 V and 0.5 mV s^−1^ scan rate resulted to be 101 F g^−1^. Integration of the discharge current (IR-drop was compensated) using the rectangular voltage waveform given in Fig. 3b yielded 115 F g^−1^ for specific capacitance (corresponding to 1.50 F cm^−2^ areal capacitance), in accordance with previous works using ACCEs^36^. The predominantly capacitive character of the underlying processes was further supported by the fact that the capacitance was uniform in open-circuit voltage from 0.18 to 1.22 V. Based on the aforementioned differential capacitance, and by considering the real impedance at 1 mHz for the 0.1-M curve in Supplementary Fig. 4b, the characteristic RC-time-constant of the ECU assembly turned out to be on the order of 100 s. The Coulombic efficiency at 1.3 V input voltage was 85–90%, still in accordance with previous works using ACCEs^36^.

In situ concentration modulation (33 mM) was measured by placing the conductivity probe in-between the first and the second ACC layer in an ACCE, namely close to the osmotic membrane section of the artificial tendril. We then estimated the concentration modulation for the whole cell treated as a single entity (lumped-parameter approximation). The estimated value was 30 mM, based on the following formula: *Q*_s_ / (*z F V*_ECF_), where *Q*_s_ is the integral charge recovered during the short-circuiting phase, *z* is the valence number of ions in the electrolyte, *F* is the Faraday constant, and *V*_ECF_ is the ECF volume. Considering the lumped-parameter approximation, the estimated value can be regarded to as a space-average. Therefore, the fact that the in situ modulation was higher than the space-average supported the choice we made for the osmotic membrane location (we aimed to expose the membrane to the largest concentration modulation).

A further concentration modulation measurement was carried out by considering a symmetric electrolyte (KCl) besides the non-symmetric one (Na_2_SO_4_), with the main aim of further investigating the observed polarity-dependent behavior shown in Fig. 3a. An ECU without artificial tendril was used. The potentiostatic driving signal was generated using an USB-powered potentiostat (CompactStat, Ivium), and the conductivity probe impedance was measured using an impedance analyzer (2273, PARSTAT). Separate ACCEs were prepared and soaked and conditioned in Na_2_SO_4_ or in KCl electrolyte. Separate calibration graphs were registered for each electrolyte. The recorded characteristic is shown in Supplementary Fig. 4e. Both electrolytes revealed a non-symmetric characteristic with respect to the input voltage polarity. In particular, with Na_2_SO_4_ electrolyte, polarization at positive voltage yielded 57% lower concentration span than negative polarization, whereas KCl electrolyte yielded 30% lower concentration span upon positive polarization. The non-symmetric characteristic also observed with the symmetric KCl electrolyte suggests a contribution by the charged functional groups such as carboxyl groups on the ACCE surface to the polarity-dependent concentration profile^37^. Indeed, carbon surface chemistry likely represents the main cause for the polarity-dependent behavior shown in Fig. 3a, since the cell was at equilibrium based on the associated timescale. We beg to also observe that, for non-symmetric electrolytes such as Na_2_SO_4_, ion mobility and charge could bring a side contribution when the cell is out of equilibrium (as it could happen even for a fully-charged cell until diffusion smears the ion concentration gradients, which are also generated by the spatial arrangement of the ACCEs layers and the artificial tendril within the cell). Finally, let us notice that the Na_2_SO_4_ concentration modulation in Supplementary Fig. 4e is higher than that one in Fig. 2j because of the smaller bulk solution volume (as obtained by removing the artificial tendril).

### ECU pressure measurement

A pressure sensor (24PCEFA6D, Honeywell) was interfaced to the artificial tendril (Supplementary Fig. 1a), and the acquired ICF pressure was digitized and synchronized (USB-6218 I/O device, National Instruments) to the input voltage.

Pressure vs. voltage was acquired at selected scan rates (Supplementary Fig. 5a). The shape of the obtained trends change when passing from lower to higher scan rates: such a transition is affected by the ~100 s RC-time-constant of the ECU (see electrochemical characterization), which introduces a delay between the input voltage and the electric potential actually occurring across the electrical double layers. The formation of dome-like trends at higher scan rates can indicate a stronger contribution of co-ion repulsion^38^ at polarity reversal. ECU charge was contextually acquired, and pressure trends were remapped vs. charge (Supplementary Fig. 5b). In the pressure vs. charge representation, the aforementioned shape transition is not observed because charge provides a time-accurate direct measure of the ions effectively immobilized.

The osmosis-driven character of the pressure response was confirmed by three additional experiments. First we considered a sodium sulfate solution at [ECF]_0_ = 0.1 M initial concentration and at *T*_0_ = 24 ± 1 °C initial temperature, and we measured ICF pressure (*P*) and ECF temperature (*T*) trends through bipolar cyclic voltammetry (Supplementary Fig. 5c). Then we induced an ICF pressure variation by a purely thermal mechanism, namely through cyclic immersion of the artificial tendril alone into [ECF] = 0.1 M solutions at different temperature (Supplementary Fig. 5d). Comparing the pressure variation per unit temperature variation in Supplementary Fig. 5c-d we could exclude the possibility of thermal effects to play a major role in the observed ICF pressure modulation. The non-thermal character of the pressure response was further highlighted by the fact that, differently from Supplementary Fig. 5c, an increase in the driving temperature in Supplementary Fig. 5d was associated with a decrease in ICF pressure (thermal expansion of the polysulfone tube likely caused an increase in the ICF-hosting volume larger than that one of the ICF itself). The osmotic character of the pressure response was further corroborated through cyclic immersion of the artificial tendril alone into solutions at *T* = 24 ± 1 °C yet having different molarity (Supplementary Fig. 5e).

### Fabrication of the artificial tendril’s effector section

The effector section of the artificial tendril was fabricated by welding together two 5 μm thick aluminum-coated polyethylene terephthalate (PET) sheets (Free Flight Supplies), with aluminized side facing outwards, so as to form a cavity open from its base (Supplementary Fig. 6a). After cutting the sheets close to the welding line, a nylon filament was inserted into the cavity (Supplementary Fig. 6b) in order to achieve a tubular cross-section (~1 mm diameter) through the subsequent shape-programming step.

For tip rotation measurement we programmed a helical shape: we coiled the previously obtained structure around a cylindrical support (Supplementary Fig. 6c), we exposed it to hot (~200 °C) air for ~10 s, then we removed the nylon filament (Supplementary Fig. 6d). For stiffness measurement we programmed a straight shape: we directly heated as described above.

The shaped tendril was then endowed with a custom fluidic connector at its base (later used to interface with the ECU). A syringe filled with ICF was interfaced to the connector and the assembly was kept in a vacuum chamber for a few minutes to degas. Upon extraction, the cavity was filled with ICF through the aforementioned syringe.

### Soft robot stiffness measurement

The bending stiffness of both natural and artificial tendrils was derived from linear beam theory^39^. Assuming a (straight) beam made of elastic material (Young modulus *E*) and having a cross-section with area moment of inertia *I*, the bending stiffness is given by *E I* (the bending stiffness accounts for both material and geometrical properties). For a beam with length *l*_f_ and clamped at one end, the bending stiffness can be computed as *F*_d_ *l*_f_^3^ / (3 *d*), where *F*_d_ denotes the force needed to induce a displacement *d* at the free end. Given a clamped tendril, we induced a displacement of the free end and we measured the corresponding reaction force, as described below.

The free end of the tendril was displaced through a custom probe (Supplementary Fig. 7a), endowed with two strain gauges (BF350, AGS-Tech. Inc.) and rigidly attached to a linear stage (M-126.CG1, Physik Instrumente). As input, the stage displacement (*d*_s_ = 2 mm) was controlled through a square-wave signal at 0.083 Hz. A half-Wheatstone-bridge was created (Supplementary Fig. 7b) and calibration was performed between its readout voltage (*U*_sg_) and the deflection force (Supplementary Fig. 7c). Calibration was also performed for the probe deflection (*d*–*d*_s_) (Supplementary Fig. 7d). For both natural and artificial tendrils we measured *d* and *F*_d_ (Supplementary Fig. 7e), so as to obtain the bending stiffness (Supplementary Fig. 7f). For the artificial tendril we also injected a volume (*V*_in_) of ICF through a syringe pump (for simplicity) so as to modulate turgor (*P*) (Supplementary Fig. 7e). This way, we could also relate bending stiffness with the injected volume (Supplementary Fig. 7f) (or with the corresponding turgor).

### Soft robot’s tip rotation measurement

The angular displacement of the soft robot tip over time was extracted by means of image processing techniques (Vision Development module in LabView, National Instruments). In particular, a visually distinctive red marker was first attached to the robot tip. Once recorded a video, the related frames (Supplementary Fig. 8a) were analyzed: after preprocessing based on hue bracketing, tendril tip orientation was obtained by using the Grayscale Value Pyramid pattern-matching algorithm (Supplementary Fig. 8b).

## Supplementary information

Supplementary Information

Description of Additional Supplementary Files

Supplementary Movie 1

Supplementary Movie 2

Supplementary Movie 3

## Data Availability

Authors can confirm that all relevant data are included in the paper and its Supplementary Information files, or are available on request from the authors.
