# Supplementary material for: A variable-stiffness tendril-like soft robot based on reversible osmotic actuation
Source: Nat Commun. 2019 Jan 21;10:344. doi: 10.1038/s41467-018-08173-y (PMC6341089; doi:10.1038/s41467-018-08173-y)
Supplement: Supplementary file 1 — Supplementary Information [file 41467_2018_8173_MOESM1_ESM.pdf]

A variable-stiffness tendril-like soft robot based on reversible osmotic actuation

Must et al.

Supplementary Information

Contains Supplementary Figures 1-8 with captions

Supplementary Figures

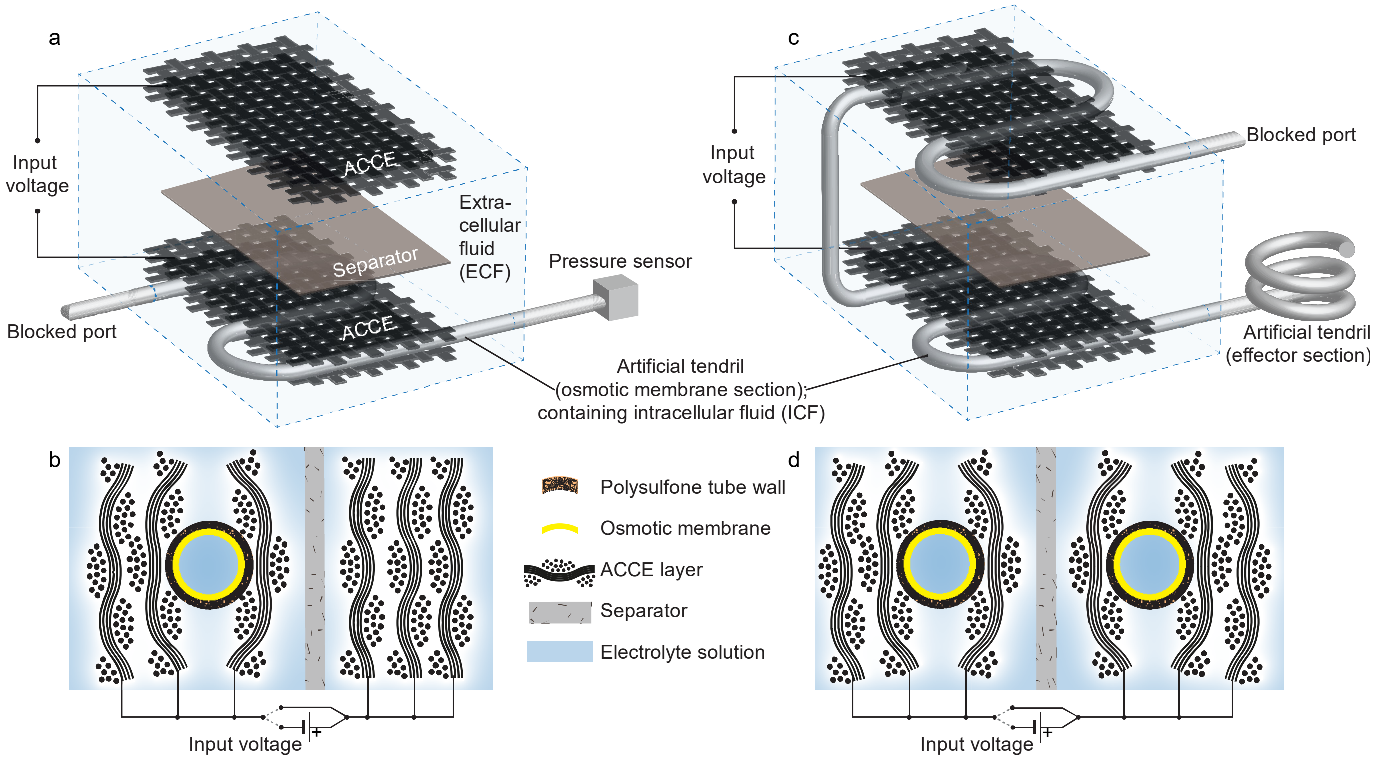

Supplementary Figure 1 | Embodiments used for development and characterization

a–b, Embodiment (schematic) used for characterizing the electroactive control unit: (**a**) only the osmotic membrane section of the artificial tendril was considered, and (**b**) it was snaking in-between a single activated carbon cloth electrode (ACCE). **c–d**, Embodiment (schematic) of the tendril-like soft robot, also used for its characterization: (**c**) the effector section of the artificial tendril was added, and (**d**) the osmotic membrane section of the tendril was snaking in-between both the (oppositely polarized) ACCEs.

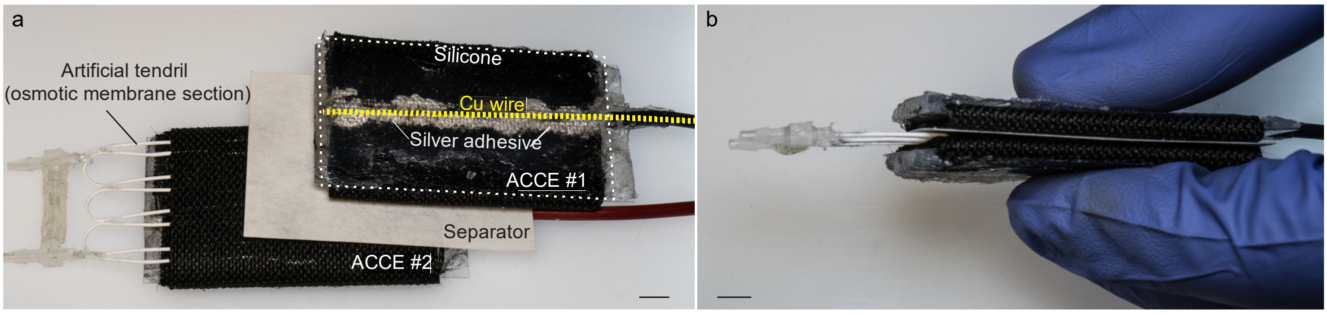

Supplementary Figure 2 | Electroactive control unit assembly

Electroactive control unit (ECU): **a**, components including the activated carbon cloth electrodes (ACCEs) and **b**, assembly. This embodiment, which corresponds to that one sketched in Supplementary Fig. 1a, was used for ECU characterization. Scale bars: 5 mm.

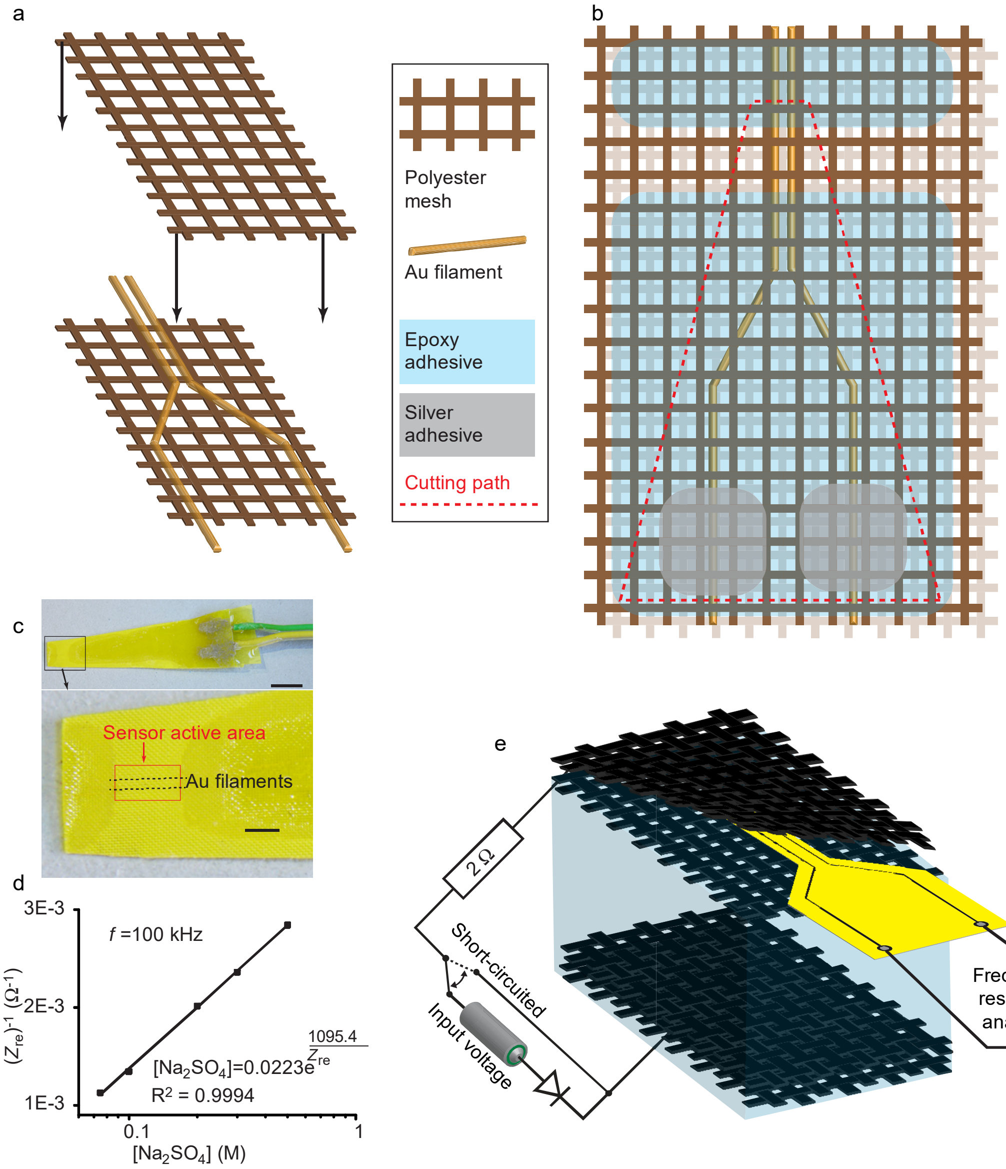

Supplementary Figure 3 | Conductivity probe for *in situ* concentration measurement

a, **b**, Fabrication (schematic) of the custom made conductivity probe: (**a**) exploded view of the polyester meshes framing the gold filaments; (**b**) top view of the assembled layers. **c**, Finished sensor (scale bars: 5 mm main image, 1 mm detail). **d**, Calibration graph for the sensor: real part of impedance (Z_{re}) versus sodium sulfate concentration, at a given frequency (f). **e**, Measurement setup (schematic).

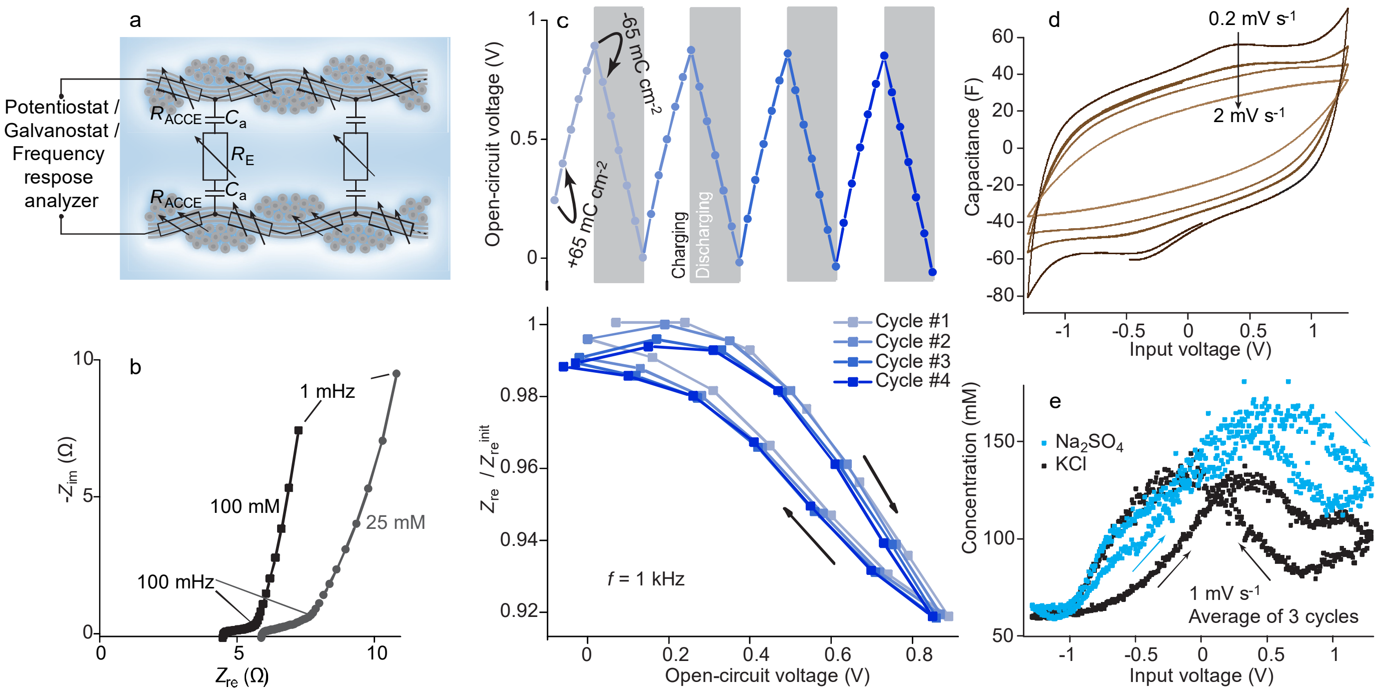

Supplementary Figure 4 | Impedance and capacitance characterization of the electroactive control unit

a, Schematic of the measurement setup, also showing the equivalent electrical circuit (R_{ACCE}: impedance of the activated carbon cloth electrode; R_E: electrolyte impedance; C_a: electric double-layer capacitance). **b**, Assembly impedance (Z_{re} and Z_{im} denoting real and imaginary component, respectively) of the electroactive control unit (ECU) at zero voltage bias for selected electrolyte concentrations in the extracellular fluid. **c**, Galvanostatic cycling of the ECU (*f* denoting frequency), showing capacitive character and voltage-dependent impedance. **d**, Capacitance versus voltage obtained by cyclic voltammetry. **e**, Non-symmetric concentration modulation with Na_2SO_4 and KCl electrolytes.

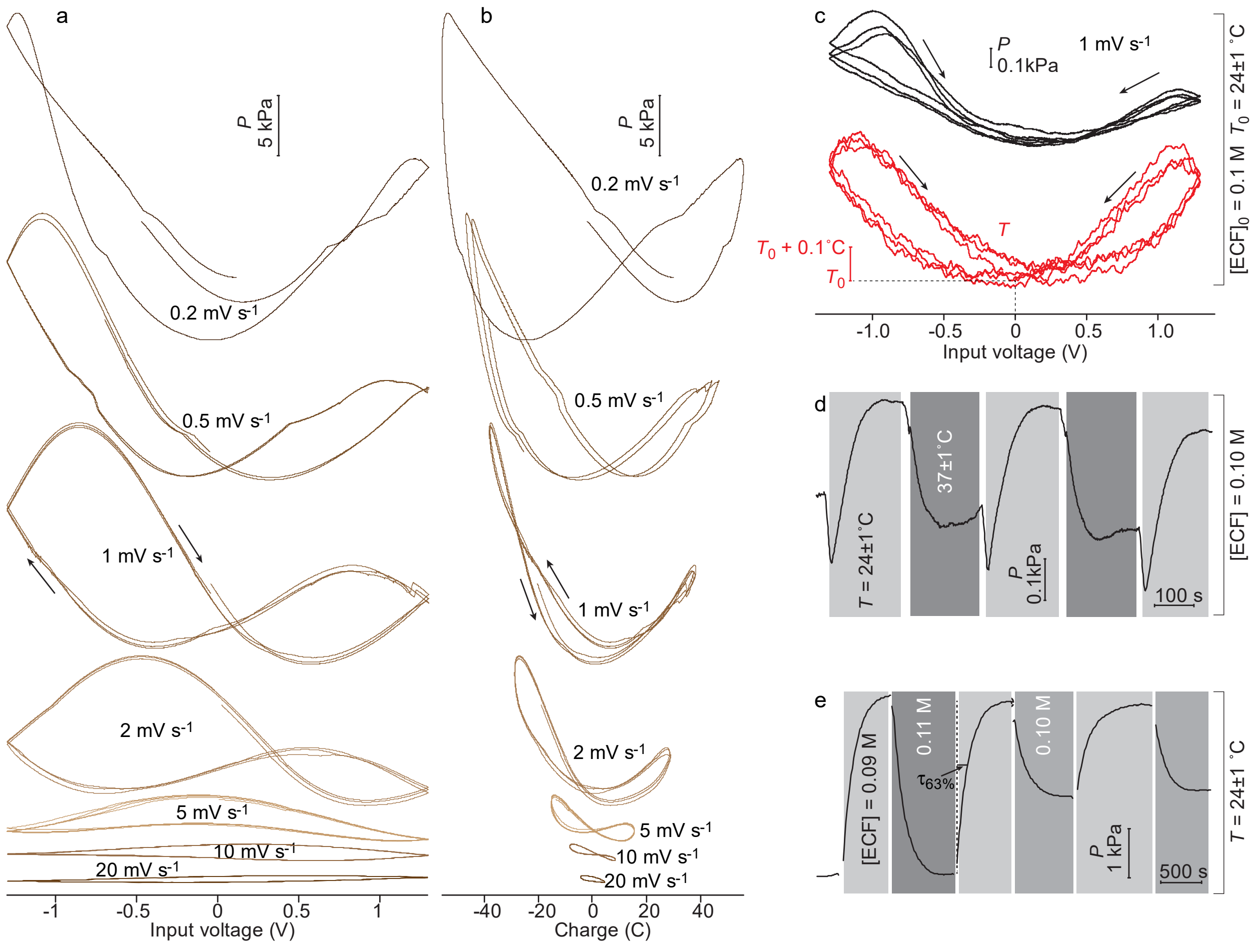

Supplementary Figure 5 |Pressure performance of the electroactive control unit assembly

a, Pressure (*P*) versus voltage at selected scan rates. **b**, Pressure versus charge at selected scan rates. **c-e**, Experiments carried out to confirm the osmosis-driven (in particular, non-thermal) character of pressure formation. (**c**) Trends of pressure in the intracellular fluid (ICF) and temperature (*T*) in the extracellular fluid (ECF), obtained through bipolar cyclic voltammetry. (**d**), ICF pressure obtained through cyclic immersion of the artificial tendril alone into solutions at different temperature. (**e**), ICF pressure obtained through cyclic immersion of the artificial tendril alone into solutions at different concentration.

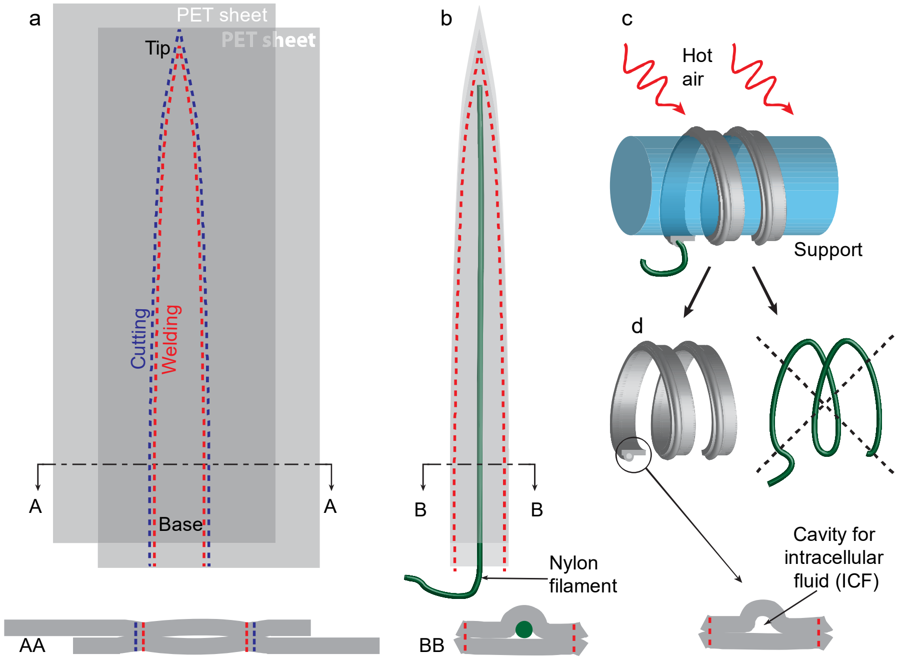

Supplementary Figure 6 | Fabrication of the artificial tendril effector

a, Polyethylene terephthalate (PET) sheets to be welded and cut so as to create a cavity. **b**, Nylon filament inserted into the cavity in order to subsequently achieve a tubular cross-section. **c**, Shape-programming: a support is used to achieve a helical effector (for a straight one the support is not needed). **d**, Tendril effector achieved after removing the filament (cavity to be filled-up with ICF).

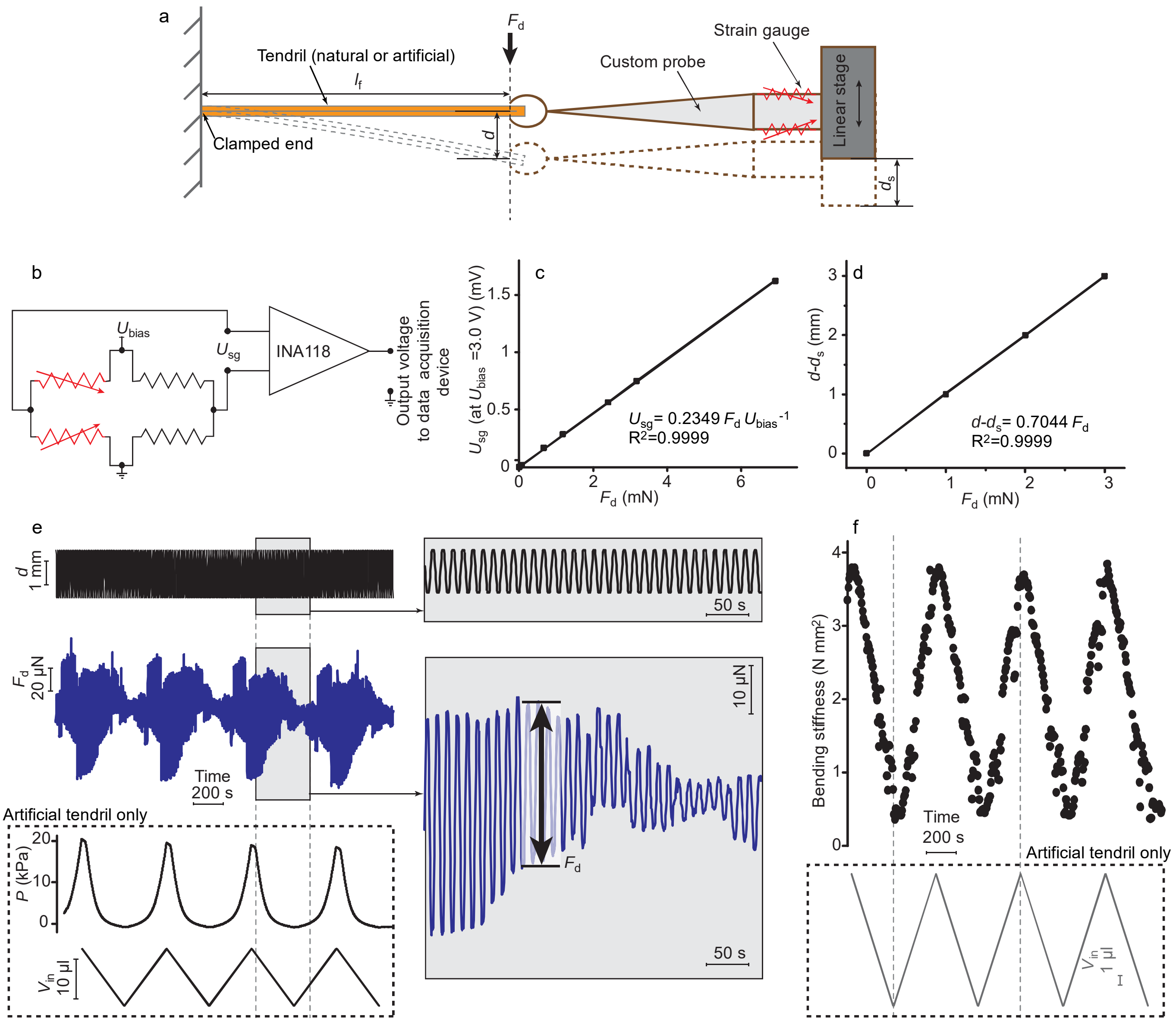

Supplementary Figure 7 | Stiffness measurement setup and procedure

a, Linear stage actively controlled to induce a displacement (*d*) of the free end of a clamped tendril through a custom probe: bending stiffness was derived by contextually recording the reaction force (F_d), also based on the length (l_f). **b-d**, Probe calibration: (**b**) half-Wheatstone-bridge showing, in particular, the readout voltage (U_{sg}); (**c**) readout voltage versus force; (**d**) deflection, also accounting for stage displacement (d_s), versus force. **e**, Illustrative recording of displacement and force versus time. For the artificial tendril, turgor (*P*) was contextually modulated by injecting a volume (V_{in}) of intracellular fluid (ICF). **f**, Derived bending stiffness versus time. For the artificial tendril, the bending stiffness can be correlated, e.g., with the injected ICF volume (or with turgor).

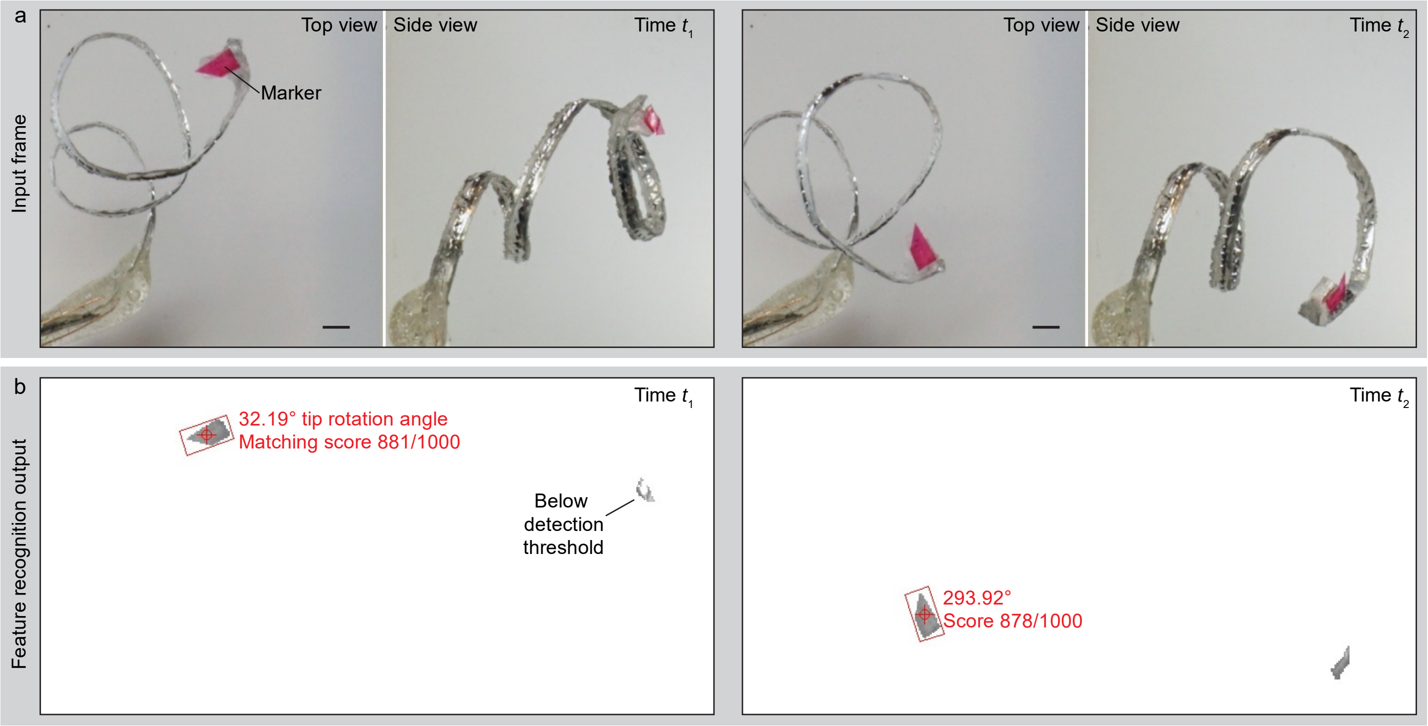

Supplementary Figure 8 | Tip rotation extraction

a, Two illustrative frames (left and right, for different values of time *t*) extracted from a video of the tendril-like soft robot coiling. **b**, Corresponding matching features extracted by image processing. Both top and side views were systematically acquired and processed, to also enhance the robustness of tip tracking. Scale bars: 1 mm.

[End of Supporting Information]
