# Supplementary material for: A variable-stiffness tendril-like soft robot based on reversible osmotic actuation
Source: Nat Commun. 2019 Jan 21;10:344. doi: 10.1038/s41467-018-08173-y (PMC6341089; doi:10.1038/s41467-018-08173-y)
Supplement: Supplementary file 2 — Description of Additional Supplementary Files [file 41467_2018_8173_MOESM2_ESM.pdf]

Description of Additional Supplementary Files

File Name: Supplementary Movie 1
Description: Main concept and working principle

File Name: Supplementary Movie 2
Description: Reversible actuation

File Name: Supplementary Movie 3
Description: Assembly of the electroactive control unit
